# What Is the Best Discipline to Predict Overall Triathlon Performance? An Analysis of Sprint, Olympic, Ironman^®^ 70.3, and Ironman^®^ 140.6

**DOI:** 10.3389/fphys.2021.654552

**Published:** 2021-05-05

**Authors:** Caio Victor Sousa, Samuel Aguiar, Rafael Reis Olher, Rafael Cunha, Pantelis Theodoros Nikolaidis, Elias Villiger, Thomas Rosemann, Beat Knechtle

**Affiliations:** ^1^Bouvé College of Health Sciences, Northeastern University, Boston, MA, United States; ^2^Department of Physical Education, University Center of Federal District – UDF, Brasilia, Brazil; ^3^Department of Physical Education, University Center of Planalto Central Apparecido dos Santos – UNICEPLAC, Brasilia, Brazil; ^4^Strength and Conditioning Laboratory, Faculty of Physical Education, University of Brasilia – UnB, Brasilia, Brazil; ^5^School of Health and Caring Sciences, University of West Attica, Athens, Greece; ^6^Institute of Primary Care, University Hospital Zurich, Zurich, Switzerland; ^7^Medbase St. Gallen Am Vadianplatz, St. Gallen, Switzerland

**Keywords:** swimming, cycling, running, half-distance, full-distance, endurance, ultra endurance

## Abstract

**Objective:** To analyze the proportion of dedication in each triathlon discipline (swimming, cycling, and running) and the importance of each separate discipline to predict overall performance of elite triathletes across different triathlon distances.

**Methods:** Data from 2015 to 2020 (*n* = 16,667) from official races and athletes in Sprint, Olympic distance, IM 70.3 (Half-Ironman distance), and IM 140.6 (Full-Ironman distance) competitions were included. The proportion of each discipline was calculated individually and compared using general linear models by event distance, sex, and performance level. Automatic linear regression models were applied for each distance considering overall performance as the dependent variable.

**Results:** A within-distance analysis showed that the best predictor for Sprint is cycling, for Olympic is swimming, for IM 70.3 is cycling, and for IM 140.6 is running. A between-distance analysis revealed that swimming is a better predictor in Olympic distance than in other triathlon distances. Cycling is a poor predictor for overall performance in IM 140.6, and the importance of running to predict overall performance is the highest in IM 140.6 and diminishes with decreasing race distance.

**Conclusion:** Each discipline represents a different relative portion and importance to predict overall performance depending on the triathlon distance. Swimming is the most important predictor discipline in Sprint- and Olympic-distance triathlon, cycling in IM 70.3, and running in IM 140.6.

## Introduction

A triathlon is a multidisciplinary sport that includes swimming, cycling, and running (Bentley et al., 2002). Triathlons started in the late 1970s with the traditional Ironman (IM) distance of 3.8 km swimming, 180 km cycling, and 42 km running (Lepers, 2008). This traditional IM distance has a total of 140.6 miles, and it will be called as IM 140.6 throughout this manuscript. Since then, new triathlon distances were created to make it less exclusive and more popular for spectators (Scott, 2004; Markus and Arimany, 2019). For instance, the traditional IM distance takes about 8 h for elite male triathletes to finish, whereas the now-popular Olympic distance (also known as standard distance) covers 1.5 km swimming, 40 km cycling, and 10 km running and takes less than 1 h 50 min for an elite male athlete to finish, and a regular single event has a larger appeal to spectators in comparison with IM (Scott, 2004; Markus and Arimany, 2019).

Later, the IM 70.3 was created to cover half the IM 140.6 distance, composed of 1.9 km swimming, 90 km cycling, and 21.1 km running (Jäckel et al., 2020). Yet another race distance with half the Olympic distance was created and named as Sprint triathlon (also known as Short triathlon), with 0.75 km swimming, 20 km cycling, and 5 km running (Markus and Arimany, 2019). There are specific differences between the distinct race distances. Shorter triathlon distances require more power and speed, whereas longer distances require more endurance and strategy (Bentley et al., 2008; Sharma and Périard, 2020). Longer triathlon distances also require extra planning for in-race hydration and nutrition (Bentley et al., 2008; Sharma and Périard, 2020). Therefore, the training and preparation for each triathlon distance involve more than just a longer training volume.

The contribution in time that each discipline has within each triathlon distance is different, which may change the training strategy to focus on a different discipline depending on the planned triathlon distance. In addition, shorter triathlon distances (e.g., Sprint- and Olympic-distance triathlon) have legal drafting in the cycling portion, which can significantly change a race dynamic. For example, faster swimmers can start cycling in a leading peloton, which causes some athletes to swim faster than planned in order to closely follow a fast pack of athletes (Gadelha et al., 2020). Different triathlon distances show a difference in race dynamics, and the contribution of each discipline varies.

The contribution of a given discipline across various distances and importance to predict overall performance can help coaches, and athletes tailor specific goals for a specific event (Figueiredo et al., 2016). It has been shown that the split disciplines contribute differently to overall race performance regarding the length of a triathlon race (Figueiredo et al., 2016; Scorcine et al., 2017). It has been shown that running was the most predictive split discipline in Olympic-distance triathlon (Gadelha et al., 2020), whereas cycling was the most predictive in IM distance triathlon (Sousa et al., 2019b). However, to the best of our knowledge, an analysis including different triathlon distances from Sprint to IM distance triathlon to determine the contribution of each discipline to overall performance in professional triathletes is missing.

Therefore, considering that the elite triathlete has the highest performance density (Lepers et al., 2013), this study aimed to investigate the proportion of dedication in each triathlon discipline and the importance of each discipline to predict overall performance across different triathlon distances to elite athletes.

## Materials and Methods

### Ethical Approval

This study was approved by the Institutional Review Board of Kanton St. Gallen, Switzerland, with a waiver of the requirement for informed consent of the participants as the study involved the analysis of publicly available data.

### Data Collection and Processing

The present analysis included data from elite athletes competing from 2015 to 2020. Only athletes ranked as professionals were considered. Data for Sprint- and Olympic-distance triathlons are publicly available and were retrieved from the World Triathlon Series (WTS) events (WTS, 2020). Data of IM 70.3 and IM 140.6 are also publicly available and were retrieved for all official IM events (Ironman^®^, 2020). All data were downloaded using a custom python script to record “event,” “event year,” “age,” “sex,” “nationality,” “swimming time,” “cycling time,” “running time,” and “overall time.”

Events with unstandardized race distances (weather conditions) were excluded from our analyses. Only official race finishers were included. No partial data were analyzed in the present study. Raw race time data in h:min:s were converted to min. The proportion of time spent in each discipline was calculated individually by the formula “discipline time/overall time × 100.” The sample was further divided into performance tertiles. The tertiles were specified by sex and race distance. Considering that all athletes included in this sample were professional triathletes, the performance tertiles were named as “fast,” “faster,” and “fastest,” being the “fastest” with lower race times, and “fast” with higher race times.

### Statistical Analysis

Data were tested for normality and homogeneity with Kolmogorov–Smirnov and Levene’s tests, respectively. Automatic regression linear models were applied for each triathlon distance to determine the importance of each discipline to predict overall performance (dependent variable). Different general linear models were applied with swimming, cycling, running, and overall performance as the dependent variables. Independent factors were “sex,” “event distance,” and “performance.” “Sex” was always included as a fixed factor with two levels (men/women), “event distance” was included as a random factor with four levels (Sprint, Olympic, IM 70.3, and IM 140.6), and “performance” was always included as a fixed factor with three levels (fast, faster, and fastest).

Performance levels were defined as race time tertiles relative to their event distance and sex. The methods of least significant difference were used for pairwise comparisons. Additional regression models were applied considering a subcohort of the fastest athletes (based on the first tertile) to determine the most influent combination of disciplines over overall triathlon performance. The standardized coefficient from each predictor (independent variables) was interpreted as a measure of importance and influence to determine the dependent variable (overall performance). Statistical significance was defined as *p* < 0.05. All statistical analyses were carried out with Statistical Software for the Social Sciences (IBM^®^ SPSS v.25, Chicago, IL, United States).

## Results

After excluding non-finishers and incomplete data, a total of 10,176 men and 6,491 women were included in the present analysis (*n* = 16,667). The number of athletes from each triathlon distance, event year, and sex is displayed in Table 1.

**Table 1:** Sample of professional triathletes from different events between 2015 and 2020.

	Men				Women			
	Sprint	Olympic	IM 70.3	IM 140.6	Sprint	Olympic	IM 70.3	IM 140.6
2015	237	298	1,042	581	221	282	631	359
2016	164	288	1,132	628	139	248	684	320
2017	138	264	970	531	120	167	580	334
2018	141	218	919	539	128	179	491	316
2019	180	180	1,195	520	167	143	696	276
2020	–	–	–	17	–	–	–	10

IM, Ironman^®^.

**Figure 1:**
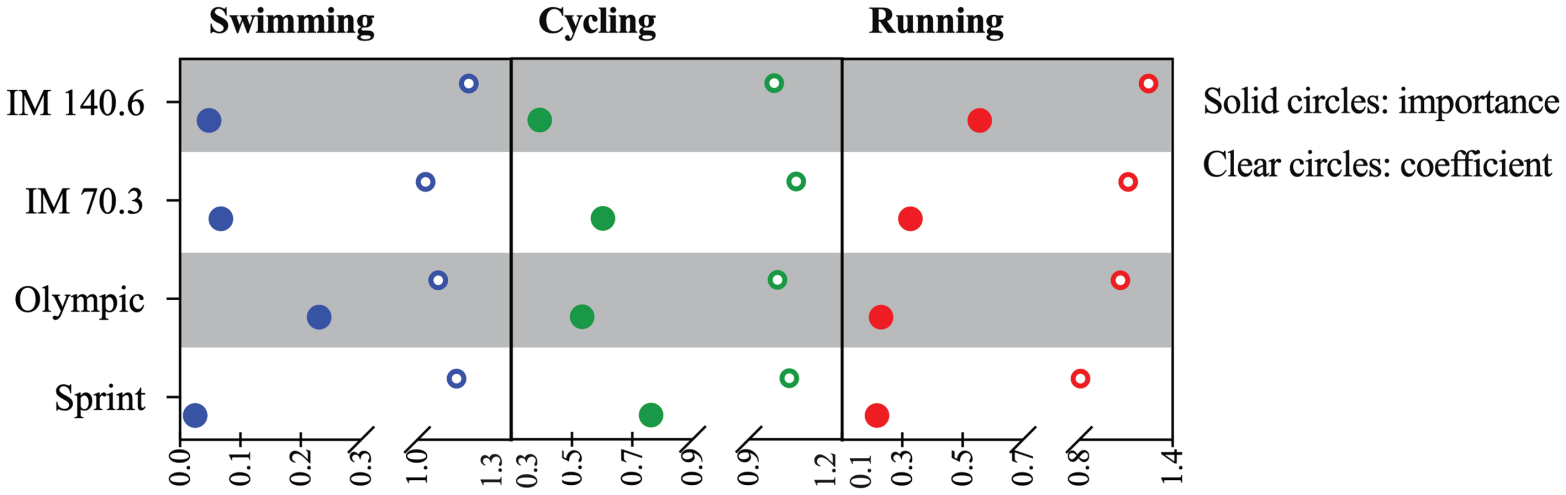
Importance (and coefficient) of swimming, cycling, and running to predict overall triathlon performance in Sprint, Olympic, IM 70.3, and IM 140.6. IM, Ironman^®^.

All four regression models showed high accuracy values to predict overall performance (Sprint: 97.2%; Olympic: 98.5%; IM 70.3: 96.4%; and IM 140.4: 92.0%). A within-distance analysis showed that the better predictor for Sprint was cycling, for Olympic was swimming, for IM 70.3 was cycling, and for IM 140.6 was running. A between-distance analysis revealed that swimming was the better predictor in Olympic-distance triathlon in comparison with all other triathlon distances, cycling was a poor predictor for overall performance in IM 140.6, and the importance of running to predict overall performance was the highest in IM 140.6 and reduced with decreasing race distance (Figure 1).

**Figure 2:**
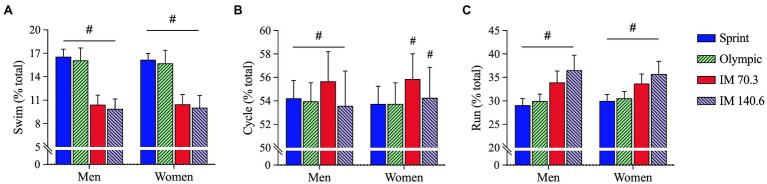
Proportion of time spent in **(A)** swimming, **(B)** cycling, and **(C)** running in different triathlon distances by elite triathletes. Data expressed as mean and standard deviation (±). IM, Ironman^®^; #, difference (*p* < 0.05) compared to other triathlon distances.

General linear models showed that “event distance” (*F* = 592.0; *p* < 0.001; *_p_η*^2^ = 0.998) and interaction “event distance × sex” (*F* = 33.3; *p* < 0.001; *_p_η*^2^ = 0.006) showed significant effects for swimming. No significant effect was identified for “sex” (*F* = 1.3; *p* = 0.336; *_p_η*^2^ = 0.299). *Post-hoc* results indicate that all triathlon distances were different from each other, with Sprint- and Olympic-distance races showing a higher proportion for swimming, in comparison with IM 70.3 and IM 140.6 (Figure 2A). Similar results were identified for cycling, with a significant “event distance” effect (*F* = 25.4; *p* = 0.012; *_p_η*^2^ = 0.962) and interaction (*F* = 31.0; *p* < 0.001; *_p_η*^2^ = 0.006). No significant effect was identified for “sex” (*F* = 0.035; *p* = 0.863; *_p_η*^2^ = 0.011). *Post-hoc* results indicate that all triathlon distances were different from each other, with IM 70.3 showing the highest proportion of cycling in comparison with all others (Figure 2B). For running, “event distance” (*F* = 61.3; *p* = 0.003; *_p_η*^2^ = 0.984) and interaction (*F* = 75.9; *p* < 0.001; *_p_η*^2^ = 0.013) showed significant effects. No significant effect was identified for “sex” (*F* = 0.101; *p* = 0.772; *_p_η*^2^ = 0.032). *Post-hoc* results indicate that all triathlon distances were different from each other, with an increasing proportion of running with increasing distance, with the Sprint distance being the lowest and IM 140.6, the highest (Figure 2C).

**Figure 3:**
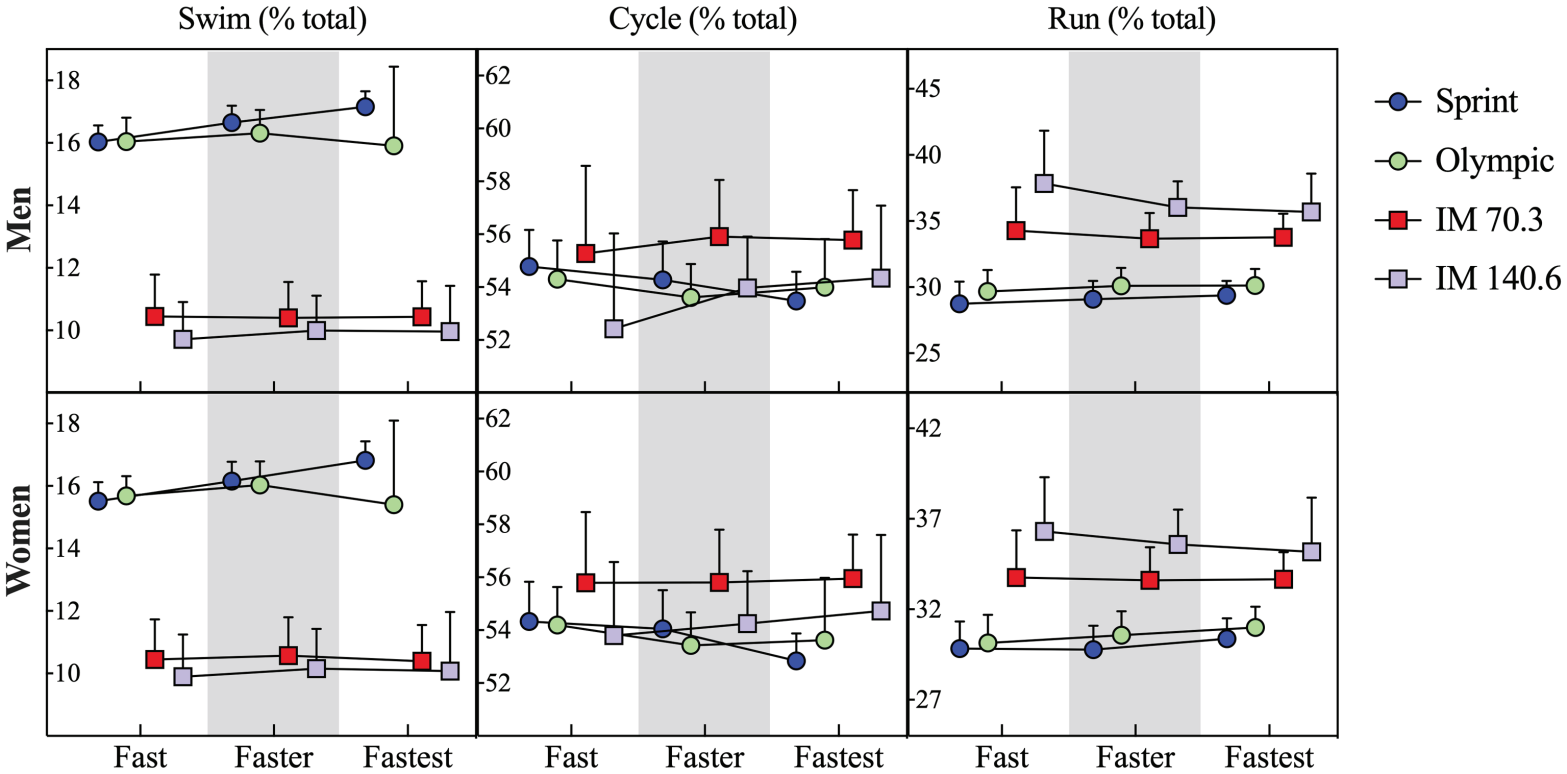
Proportion of time spent in swimming, cycling, and running in different triathlon distances by elite men and women triathletes in different performance tertiles. Data expressed as mean and standard deviation (±). IM, Ironman^®^.

As expected, the significant effects for “performance” and “event distance” were identified for both men and women (*p* < 0.001). Pairwise comparison confirmed the “fastest” triathletes with the lowest race times, and IM 140.6 with highest race times (Table 2). The models considering “performance” and “race distance” as independent factors showed that “event distance” had a significant effect over the proportion of swimming (men: *F* = 575.2; *p* < 0.001; *_p_η*^2^ = 0.997; women: *F* = 318.2; *p* < 0.001; *_p_η*^2^ = 0.994), cycling (men: *F* = 10.5; *p* = 0.008; *_p_η*^2^ = 0.840; women: *F* = 17.1; *p* = 0.002; *_p_η*^2^ = 0.895), and running (men: *F* = 65.8; *p* < 0.001; *_p_η*^2^ = 0.971; women: *F* = 104.1; *p* < 0.001; *_p_η*^2^ = 0.981) disciplines. Conversely, “performance” was not significant for any triathlon discipline (Figure 3).

**Table 2:** Overall performance (minutes) of men and women across different triathlon distances by performance tertiles.

		Sprint	Olympic	IM 70.3	IM 140.6
Men	Fast	57.4 ± 1.5	116.4 ± 3.0	270.1 ± 25.8	596.1 ± 58.3
	Faster	54.8 ± 0.5	110.8 ± 1.1	244.5 ± 4.0	529.5 ± 9.8
	Fastest	52.9 ± 0.8	107.0 ± 1.8	230.1 ± 5.8	493.5 ± 15.9
Women	Fast	63.2 ± 1.5	128.2 ± 3.6	294.2 ± 14.4	633.8 ± 36.5
	Faster	60.5 ± 0.6	122.0 ± 1.2	272.3 ± 4.1	581.9 ± 9.7
	Fastest	58.2 ± 0.8	117.2 ± 2.5	256.4 ± 6.4	544.5 ± 18.5

Data expressed as mean ± standard deviation.

IM, Ironman^®^.

The subcohort with the fastest athletes had all models significantly (*p* < 0.001; *R*^2^ > 0.9). The analyses showed that the combination “Bike + Run” is the most influent combination in all triathlon distances, and the least influent is “Swim + Run” in Sprint, IM 70.3, and IM 140.6. The combination “Swim + Bike” is the least influent in Olympic triathlon. See Table 3 for details.

**Table 3:** Influence of the discipline combination over overall performance in the fastest triathletes in Sprint, Olympic, IM 70.3, and IM 140.6.

	Sprint	Olympic	IM 70.3	IM 140.6
Swim + Bike	0.316	0.346	0.364	0.374
Swim + Run	0.282	0.350	0.264	0.331
Bike + Run	0.446	0.465	0.460	0.468

Data expressed in standardized coefficient (beta).

IM, Ironman^®^.

## Discussion

This is the first study to analyze the proportion of time spent in each triathlon discipline and its importance to predict overall performance across different triathlon distance events using a large sample. The main findings of this study were (1) swimming was the better predictor for overall performance in Olympic-distance triathlon, and swimming also represented a larger portion in Sprint- and Olympic-distance triathlon relative to total race time in comparison with IM 70.3 and IM 140.6; (2) cycling represented the larger proportion relative to overall race time and was also the better predictor for overall race time in IM 70.3; and (3) the running discipline proportion and the ability to predict overall performance increased with increasing race distance.

### Swimming as the Best Predictor in Olympic-Distance Triathlon

The first important finding was that swimming was the better predictor for overall performance in Olympic-distance triathlon. Swimming was the discipline representing the smallest portion in all triathlon distances (Scorcine et al., 2017), but its importance was different depending on the triathlon event. In draft-legal triathlons (i.e., Sprint and Olympic), a slow swim may result in a slower peloton or a lonely cycling portion. Cycling within a peloton requires lower oxygen uptake and lower heart rate for a higher power output (Edwards and Byrnes, 2007). Thus, athletes cycling within a fast peloton can save energy while drafting and get into better conditions in the running portion (Ofoghi et al., 2016). In IM 70.3 and IM 140.6, where drafting is not allowed, the athlete does not need to push harder than planned to stay within the first pack because the cycling portion has no leading or chasing pelotons (Barbosa et al., 2019; Jäckel et al., 2020).

Nevertheless, our results showed that swimming does not represent a good predictor of overall performance in Sprint triathlons. Although Sprint triathlons are draft-legal races and swimming represents a relatively high portion of the race, the shorter swimming distance (an average of 9.7 min) may allow athletes to swim closer together – which is reflected in the lowest data dispersion across disciplines and triathlon events, reducing the swimming importance to overall performance. Furthermore, previous studies found that, compared to swimming alone, draft swimming reduces energy expenditure which allows a stable stroke frequency and length for a better power output (Chollet et al., 2000).

### Cycling as the Predictor in Sprint and Half-Ironman Distance Triathlon

A second important finding was that cycling was the better predictor for overall race time in IM 70.3. Conversely to the swimming portion, cycling is the larger portion in all triathlon distances, but the best cyclist does not always win the race. For Olympic triathlons, our results corroborate previous research showing that despite being the larger portion, cycling was not the most determinant discipline for overall performance (Ofoghi et al., 2016), mostly due to drafting rules making most athletes cycle at a similar pace in large pelotons. On the other hand, for Sprint triathlon, cycling was the most important discipline to predict overall race time. For IM 70.3 and IM 140.6, our results showed that cycling was not a good predictor for overall race time. IM 70.3 and IM 140.6 are events where drafting is prohibited, allowing athletes to pace according to their own ability and strategy (Barbosa et al., 2019; Jäckel et al., 2020). Though athletes hardly have extraordinary cycling performances because of the running portion that follows cycling, athletes cycle fast enough only to keep sight of the leaders but save enough energy for a competitive run (Angehrn et al., 2016; Knechtle et al., 2019).

Our results are different from the previous research showing that cycling was the best predictor for overall race time in IM 140.6 (Sousa et al., 2019b). However, that study only included the very best male performances (under 8 h), which represented only a small portion of elite athletes (<100). Other important aspects to be accounted regarding the amount of time in each discipline in different triathlon distances included hydration, nutrition, and training (Jeukendrup et al., 2005; Etxebarria et al., 2019; Sousa et al., 2019a). The cycling portion represents not only the highest amount of time spent in a triathlon, but it is also very strategic to eat and hydrate after the swimming portion in which athletes usually do not eat or drink. During cycling, athletes also have to eat and drink while preparing for the running portion, which is the discipline with most dropouts, stomach discomforts being the main reason in IM 140.6 (Pfeiffer et al., 2012). Discipline performance might also relate to the distribution of training volume among the disciplines (Knechtle et al., 2015; Etxebarria et al., 2019).

### Running as the Predictor in Ironman Distance Triathlon

Both swimming and cycling proportions seemed to be affected by race draft regulations, changing the dynamics of the race and the importance of each discipline. However, the running split seemed to follow the expected trend in longer distances with a slowing down in pace (Knechtle et al., 2019). It is noteworthy that a slower running split should account for more than just the in-race running distance (i.e., 5 km, 10 km, 21.1 km, and 42.195 km), but also the greater physical demand of the previous disciplines, exposure to heat (in some events), dehydration, carbohydrate reposition, and psychological distress (Dallam et al., 2005).

Our results corroborate previous results showing that running was of intermediate importance to predict overall race time in Olympic and IM 70.3 (Ofoghi et al., 2016; Jäckel et al., 2020). This study is, however, the first to show a similar trend in Sprint triathlon. Conversely, this is also the first study to show that running was the better predictor of overall race time in IM 140.6. In any triathlon distance, running is the discipline to be performed with previous swimming and cycling fatigue (O’Toole and Douglas, 1995). Our results show that, in IM 140.6, this accumulated fatigue significantly increases the importance of running to overall performance. An explanation of this discovery might be that running is weight-bearing locomotion in contrast to the other two disciplines in which body weight is supported by either the bicycle or the water. Accordingly, a longer duration of a running event might result in increased fatigue related to body weight.

### Sex Differences

The present analyses did not find any significant effect of sex over the contribution of each discipline (%) to overall performance in all four triathlon distances. The literature is consistently showing that males are faster than females across all triathlon distances (Lepers, 2019; Gadelha et al., 2020), and in all three triathlon disciplines (Lepers et al., 2013; Lepers, 2019). Nevertheless, our results show that performance difference does not change the relative contribution and importance of each discipline to overall performance. However, it is noteworthy that women are becoming increasingly faster in ultra-triathlons (Sousa et al., 2019c), closing the performance sex gap in long-distance open-water swimming events (Nikolaidis et al., 2018).

### Practical Applications and Limitations

As for practical applications, athletes who are very good swimmers have a better chance of success in an Olympic-distance triathlon for two main reasons: (1) swimming represents a larger proportion of the race in comparison with longer triathlon distances (i.e., IM 70.3 and IM 140.6) and (2) faster swimming can put the athlete within the faster cycling peloton. Additionally, athletes whose best discipline is cycling have better chances of success in Sprint and IM 70.3, since cycling represents the longer portion relative to other distances, and for IM 70.3, drafting is illegal. Athletes whose best discipline is running might do better in longer races, especially in IM 140.6. Finally, for coaches and recreational athletes, this study becomes important to be able to direct the training of athletes so that they can prioritize or increase the potential of the modalities according to the type of race that the athlete plans to compete or specialize.

Our results require caution when applying to non-professional athletes (i.e., recreational athletes and age group athletes), since all professional athletes are relatively good in all triathlon disciplines, whereas most non-professional athletes have one or more disciplines that could be considered as a weakness. Additionally, transition times were summed to cycling and running in this analysis, and transition can have independent importance to overall performance, especially in shorter races. A limitation is that drafting is allowed in cycling in the shorter triathlon races (i.e., Sprint- and Olympic-distance triathlon). Additionally, in-race factors were not considered for this analysis and should be further explored in future studies. For instance, Short- and Olympic-distance triathlons usually take place in short bike course, requiring an elevated power output variability when existing numerous turns with a need to remain with the peloton. However, in IM triathlons, the courses usually have less turns allowing a more stable power output.

## Conclusion

In conclusion, each discipline represents a different relative portion and importance to predict overall race time depending on the triathlon distance. Swimming represents a larger portion in Sprint and Olympic relative to total race distance in comparison with longer races (IM 70.3 and IM 140.6) and is also a better predictor for overall race time in Olympic-distance triathlons. Cycling represents the larger proportion relative to total race distance and is the better predictor for overall race time in IM 70.3. The proportion of the running discipline to overall race time and its ability to predict overall performance is low in Sprint triathlons and increases with the increasing race distance, being the better overall performance predictor in IM 140.6.

## Data Availability Statement

The raw data supporting the conclusions of this article will be made available by the authors, without undue reservation.

## Author Contributions

All authors contributed to the study conception and design. Material preparation and data collection were performed by EV, PN, and BK. Data analysis was performed by CS. Data interpretation was conducted by CS, SA, RO, and RC. The first draft of the manuscript was written by CS and BK. All authors commented on previous versions of the manuscript. All authors contributed to the article and approved the submitted version.

### Conflict of Interest

The authors declare that the research was conducted in the absence of any commercial or financial relationships that could be construed as a potential conflict of interest.
